# Integrating Semi-supervised and Supervised Learning Methods for Label Fusion in Multi-Atlas Based Image Segmentation

**DOI:** 10.3389/fninf.2018.00069

**Published:** 2018-10-10

**Authors:** Qiang Zheng, Yihong Wu, Yong Fan

**Affiliations:** ^1^Department of Radiology, Perelman School of Medicine, University of Pennsylvania, Philadelphia, PA, United States; ^2^School of Computer and Control Engineering Yantai University, Yantai, China; ^3^National Laboratory of Pattern Recognition, Institute of Automation, Chinese Academy of Sciences, Beijing, China

**Keywords:** multi-atlas, image segmentation, hippocampus, random forests, label propagation

## Abstract

A novel label fusion method for multi-atlas based image segmentation method is developed by integrating semi-supervised and supervised machine learning techniques. Particularly, our method is developed in a pattern recognition based multi-atlas label fusion framework. We build random forests classification models for each image voxel to be segmented based on its corresponding image patches of atlas images that have been registered to the image to be segmented. The voxelwise random forests classification models are then applied to the image to be segmented to obtain a probabilistic segmentation map. Finally, a semi-supervised label propagation method is adapted to refine the probabilistic segmentation map by propagating its reliable voxelwise segmentation labels, taking into consideration consistency of local and global image appearance of the image to be segmented. The proposed method has been evaluated for segmenting the hippocampus in MR images and compared with alternative machine learning based multi-atlas based image segmentation methods. The experiment results have demonstrated that our method could obtain competitive segmentation performance (average Dice index > 0.88), compared with alternative multi-atlas based image segmentation methods under comparison. Source codes of the methods under comparison are publicly available at www.nitrc.org/frs/?group_id=1242.

## Introduction

In recent years, multi-atlas based image segmentation (MAIS), as an automatic medical image segmentation technique, has been widely adopted in medical image segmentation studies (Hao et al., 2012a,b,c, 2014; Zhu et al., 2015, 2016, 2017; Zheng and Fan, 2018). Most typical MAIS methods consist of two components: atlas image registration and atlas label fusion. Particularly, the atlas image registration component aligns atlas images and their segmentation labels to an image to be segmented, and then the aligned segmentation labels are fused to obtain a segmentation result for the image to be segmented by the atlas label component. Besides improving the image registration component (Hao et al., 2012b; Alven et al., 2016; Doshi et al., 2016; Alchatzidis et al., 2017), many MAIS methods have focused on improving the label fusion component (Rohlfing et al., 2004; Coupé et al., 2011; Han, 2013; Wang et al., 2013; Hao et al., 2014; Amoroso et al., 2015; Roy et al., 2015; Wu et al., 2015; Zhu et al., 2015, 2017; Doshi et al., 2016; Giraud et al., 2016; Zhang et al., 2017; Zu et al., 2017; Yang and Fan, 2018a,b). In particular, majority voting (MV) might be the simplest label fusion method (Rohlfing et al., 2004), and more sophisticated label fusion strategies have been built upon a nonlocal patch-based label fusion strategy (Coupé et al., 2011), such as metric learning (Zhu et al., 2017), joint label fusion (Wang et al., 2013), and dictionary learning (Roy et al., 2015; Yang and Fan, 2018a). A pattern recognition framework has also been developed to solve the label fusion problem (Hao et al., 2012c, 2014; Han, 2013). In the pattern recognition based label fusion framework, image patches of the registered atlases and their segmentation labels are used as training data to build pattern classification models using support vector machines (SVM) (Hao et al., 2012c, 2014), linear regression model (Zhu et al., 2015), artificial neural networks (ANNs) (Amoroso et al., 2015), or random forests (RF) (Han, 2013), and then the trained pattern classification models are used to predict segmentation labels of image patches in the image to be segmented.

Although the existing label fusion methods differ in many aspects, they typically implement the label fusion for different voxels independently without taking into consideration spatial consistency among voxels of images to be segmented. Since imaging noise often presents in biomedical images, it may obtain degraded performance to segment image voxels independently. Furthermore, the pattern recognition label fusion methods might be hampered by discrepancy between atlas images and images to be segmented, which cannot be fully eliminated by the image registration, particularly image intensity differences (Li et al., 2010; Coupé et al., 2011; Li and Fan, 2012). These problems have been addressed in different studies for medical image segmentation problems (Li et al., 2010; Coupé et al., 2011; Li and Fan, 2012; Koch et al., 2014). However, a unified solution is desirable for segmenting images in the MAIS framework.

In this study, we develop a novel MAIS method by integrating a semi-supervised label propagation (SSLP) method and a supervised random forests (RF) classification method in the MAIS framework (Breiman, 2001; Zhou et al., 2004), aiming to achieve improved image segmentation performance. Particularly, after atlas images and their segmentation labels are registered to an image to be segmented, a local RF classification model is built for every image voxel to be segmented upon its neighboring image patches of the registered atlas images in the MAIS framework to obtain a probabilistic image segmentation map (Hao et al., 2012c, 2014; Han, 2013). Then, the probabilistic image segmentation map is refined by a semi-supervised label propagation method that propagates reliable segmentation information within the image to be segmented, under a constraint of local and global image intensity consistency (Zhou et al., 2004; Coupé et al., 2011; Li and Fan, 2012). An information-balance-weighting scheme is proposed to propagate the reliable image segmentation within the image to be segmented itself, rather than propagating information among the target image and the atlas images (Koch et al., 2014), which may suffer from image intensity inconsistency across images. We have validated the proposed method for segmenting the hippocampus in magnetic resonance imaging (MRI) scans based on segmentation labels provided by the EADC–ADNI (European Alzheimer's Disease Consortium and Alzheimer's Disease Neuroimaging Initiative) harmonized segmentation protocol (http://www.hippocampal-protocol.net; Boccardi et al., 2015). Comparison results with alternative MAIS methods have demonstrated that the proposed method could achieve competitive segmentation performance. Preliminary results of this study have been reported in a conference paper (Zheng and Fan, 2018). Source codes of the methods under comparison and segmentation evaluation metrics are publicly available at www.nitrc.org/frs/?group_id=1242.

## Materials and methods

### Imaging data and hippocampus atlases

In this study, we used publicly available imaging data to develop and validate our method. Particularly, magnetic resonance imaging (MRI) scans were obtained from the Alzheimer's Disease Neuroimaging Initiative (ADNI) database (adni.loni.usc.edu/). The ADNI was launched in 2003 as a public-private partnership, led by Principal Investigator Michael W. Weiner, MD. The primary goal of ADNI has been to test whether serial magnetic resonance imaging (MRI), positron emission tomography (PET), other biological markers, and clinical and neuropsychological assessment can be combined to measure the progression of mild cognitive impairment (MCI) and early Alzheimer's disease (AD). The ADNI MRI scans were acquired using a sagittal 3D MP-RAGE T1-w sequence (TR = 2,400 ms, minimum full TE, TI = 1,000 ms, FOV = 240 mm, voxel size of 1.25 × 1.25 × 1.2 *mm*^3^; Jack et al., 2008). For up-to-date information, see www.adni-info.org.

To validate our method for segmenting the hippocampus, a subset of the ADNI MRI scans with manual segmentation labels of the hippocampus was obtained from the EADC-ADNI harmonized segmentation protocol project (www.hippocampal-protocol.net; Boccardi et al., 2015). In particular, the hippocampus segmentation labels of 135 subjects are available, consisting 100 subjects from a preliminary release and 35 subjects from a final release. In the present study, we downloaded MRI scans and their hippocampus labels in NIFTI format. In the preliminary release dataset, 002_S_0938's bilateral hippocampus labels missed several slices. In the final release dataset, MRI scans of 4 subjects (007_S_1304, 002_S_4121, 029_S_4279, and 136_S_0429) are not matched with their corresponding label images due to image format conversion. The mismatch problem was corrected by reorienting the hippocampus label images. Finally, we obtained 99 subjects and 35 subjects from preliminary release and final release, respectively, with each subject having both MRI scan and its corresponding hippocampus label. The data from the final release were used as atlas images, and the data from the preliminary release were used as test data in our study. Basic demographic data of these subjects are summarized in Table 1.

**Table 1:** Demographic information.

	**Subject size**	**Males/ Females**	**Age (years): mean ±std**
Normal controls (NC)	44	22/22	76.18 ± 7.45
Mild cognitive impairment (MCI)	46	27/19	74.70 ± 8.10
Alzheimer's disease (AD)	45	21/24	74.45 ± 8.10

### Multi-atlas based image segmentation by integrating semi-supervised label propagation and random forests

Our method, referred to as RF-SSLP, consists of an MAIS method for generating a probabilistic segmentation result using a RF classification method and a SSLP method for computing reliable image segmentation.

#### Local label fusion based on random forest

Given a target image *I* and *N* registered atlas images and their labels *A*_*i*_ = (*I*_*i*_, *L*_*i*_), *i* = 1, ⋯, *N*, where *I*_*i*_ and *L*_*i*_ are the *i*^*th*^ atlas and label images, respectively, random forest models are built to generate a probabilistic segmentation result of the target image in a pattern recognition framework (Hao et al., 2014). Particularly, a pattern recognition model is built for each voxel to be segmented based on image features computed from image patches (Coupé et al., 2011).

In order to segment each voxel in the target image, a 3D image patch consisting of its neighboring voxels with size (2*r*_*s*_ + 1) × (2*r*_*s*_ + 1) × (2*r*_*s*_ + 1) is identified to compute image features for characterizing the voxel under consideration, where *r*_*s*_ is the image patch's radius. Particularly, image intensity values of the image patch are first normalized by subtracting its mean value and followed by dividing its standard deviation, then a set of texture filters are applied to the normalized image patch for computing image texture features, including the first and second order difference filters, 3D Hyperplane filters, 3D Sobel filters, Laplacian filters, and Range difference filters are utilized to extract features, and finally the texture features and image intensity values are concatenated to form a feature vector (Hao et al., 2014). The same image feature extraction procedure is used to compute image features for each voxel of both the target image and the atlas images.

The image features extracted from the atlas images with their segmentation labels are used as training data for building pattern classification models for image voxels to be segmented. Particularly, a pattern recognition model can be built for each voxel of the image to be segmented based on its neighboring voxels of the atlas images (Hao et al., 2014). Given a voxel *x* to be segmented in the target image, voxels of the atlas images in its neighborhood *N*(*x*) with (2*r*+1) × (2*r*+1) × (2*r*+1) are used as training samples, where *r* is the neighborhood size for building training data. For each of the training voxels, image features are computed as aforementioned, and we obtain (2*r*+1)^3^ × *N* training samples $\left\{\left({\vec{f}}_{i,j},\;l_{i,j}\right)|i=1,\cdots \;,N,\;j\in N\left(x\right)\right\}$, where ${\vec{f}}_{i,j}$ is a feature vector with label *l*_*i, j*_ ∈ {1, 0} with 1 indicating the hippocampus and 0 indicating background. Based on the training samples, a RF classification model is trained for predicting the segmentation label of the voxel under consideration. To build the classification model on balanced training samples, we select the same number (*k*) of positive and negative samples that are most similar to the voxel to be segmented, to train the classification model (Hao et al., 2014). The similarity between voxels is measured by Euclidean distance between their image features.

The random forest classification model is an ensemble of classification trees that are built upon the training data using randomly sampling, with 2 parameters: *N*_*Tree*_ (the number of trees) and *N*_*Split*_ (the number of predictors sampled for splitting at each node; Breiman, 2001). Once *N*_*Tree*_ classification trees are trained, they are applied to image features *f*_*x*_ of voxel *x* to predict its segmentation label:

(1) $$\begin{array}{r} p\left(f_{x}\right)=\frac{1}{N_{Tree}}\sum_{i=1}^{N_{Tree}}T_{i}\left(f_{x}\right) \end{array}$$

where *T*_*i*_ is the *ith* tree with a classification result of 1 or 0, and *p* is a probabilistic segmentation result. Applying the voxelwise classification model to an image to be segmented, we obtain a probabilistic segmentation map. The probabilistic segmentation map could be binarized using a threshold of 0.5 to obtain a binary segmentation image. Therefore, in addition to a binary segmentation result for each voxel of the image to be segmented, we also obtain a probabilistic segmentation map for the image to be segmented. Since a segmentation result with probabilistic value close to 0.5 might be unreliable and the segmentation results might be degraded by imaging noise since they are obtained for different voxels separately, we adopt a semi-supervised label propagation method to refine the probabilistic segmentation map (Zhou et al., 2004; Li et al., 2010; Coupé et al., 2011; Li and Fan, 2012).

#### Semi-supervised label propagation for the hippocampus segmentation

A graph theory based label propagation method is adopted to refine the probabilistic image segmentation map (Zhou et al., 2004). Particularly, to refine the probabilistic segmentation map with *n* voxels under a constraint of local and global image consistency, the label propagation obtains a new segmentation map by minimizing

(2) $$\begin{array}{r} E\left(L\right)=L^{T}\left(I-S\right)L+{\alpha \left(L-P\right)}^{T}\left(L-P\right), \end{array}$$

where *P* is an *n* × 2 matrix for encoding foreground and background of the probabilistic segmentation map to be refined, respectively, with *p*_*i*, 1_ = *max*(2 × (*p*(*f*_*i*_) − 0.5), 0), *p*_*i*, 2_ = *max*(2 × (0.5 − *p*(*f*_*i*_)), 0), *i* = 1, …, *n*, *p*(*f*_*i*_) is the probabilistic segmentation result obtained by the Local label fusion based on random forest, *L* is an *n* × 2 matrix for encoding foreground and background of the refined segmentation map, *S* is a symmetric normalized Laplacian matrix, *I* is an identity matrix, and α is a parameter. Particularly, *S* plays an important role in the label propagation in that it characterizes similarity among different voxels. In our study, image similarity between two voxels *x* and *y* is defined by

(3) $$W_{xy}=\{\begin{array}{c} \exp\left(-\frac{{\left\|I_{x}-I_{y}\right\|}^{2}}{\sigma^{2}}\right),x\neq y\\ 0,\;x=y \end{array},$$

where *I*_*x*_ and *I*_*y*_ are image intensity values of voxels *x* and *y*, respectively, and σ is a parameter. Given a pairwise similarity matrix, *W*, between all voxels defined by Equation (3),

(4) $$\begin{array}{r} S=D^{-1/2}WD^{-1/2}, \end{array}$$

where *D* is a diagonal matrix with its diagonal element equal to the sum of the corresponding row of *W*.

The optimization problem of Equation (2) can be solved iteratively as follows (Zhou et al., 2004):

(5) $$\begin{array}{r} L^{n+1}=\left(1-\beta \right)SL^{n}+\beta P, \end{array}$$

where *n* is the number of iteration steps, and 0 < β < 1 is a trade-off parameter related to α.

**Figure 1:**
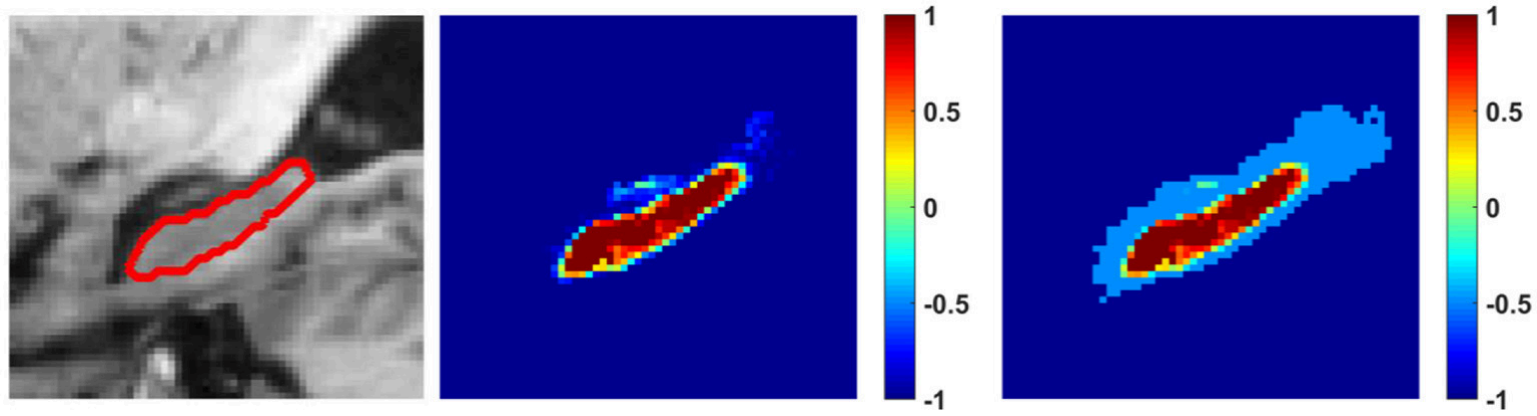
An example image slice with the hippocampus boundary (left), its probabilistic segmentation map obtained by the random forest classification model (middle), and its probabilistic segmentation map with balanced label information (right). The colorbars indicate segmentation probability values or balanced reliable label information for background (−1~0) and hippocampus (0~1). The threshold *T* was 0.5.

To relieve impact of unreliable image segmentation results on the label propagation, the probabilistic segmentation map is weighted by reliable segmentation information, referred to as information-balance-weighting. Particularly, the reliable segmentation result is determined by a threshold *T*. Since the number of background voxels is typical larger than the number of the foreground voxels, to balance the background and foreground label information in the label propagation, the reliable background label information is enhanced as

(6) $$p_{i,2}^{*}=\{\begin{array}{c} \max\left(\frac{N_{f}}{N_{b}}p_{i,2},T\right),\;p_{i,2}>T\\ p_{i,2},\;p_{i,2}\leq T \end{array},$$

where *N*_*f*_ is the number of voxels with reliable foreground (hippocampus) segmentation labels, *N*_*b*_ is the number of voxels with reliable background segmentation labels, and *T* is the threshold for determining the reliable segmentation result. It is worth noting that max(·, *T*) guarantees that the reliable $p_{i,2}^{*}$ remains no smaller than the threshold *T* after the information-balance-weighting. Then, *P* is normalized by updating *p*_*i*, 1_ with $p_{i,1}\left(p_{i,1}>T\right)=p_{i,1}\left(p_{i,1}>T\right)/\bar{p_{i,1}\left(p_{i,1}>T\right)}$ and updating *p*_*i*, 2_ with $p_{i,2}\left(p_{i,2}^{*}>T\right)=p_{i,2}^{*}\left(p_{i,2}^{*}>T\right)/\bar{p_{i,2}^{*}\left(p_{i,2}^{*}>T\right)}$, where *p*_*i*, 1_ encodes the foreground segmentation label, *p*_*i*, 2_ encodes the background segmentation label, and $\bar{p_{i,1}\left(p_{i,1}>T\right)}$ and $\bar{p_{i,2}^{*}\left(p_{i,2}^{*}>T\right)}$ are mean values of the reliable foreground and background labels, respectively. Finally, a refined segmentation coding matrix *L* obtained by updating the normalized segmentation coding, as formulated by Equation (5), and a binary refined segmentation map is obtained by assigning voxels as foreground if *l*_*i*, 1_ > *l*_*i*, 2_, *i* = 1, …, *n* or background otherwise. An example image slice, its probabilistic segmentation map obtained by the random forest classification model, and its balanced label information are shown in Figure 1.

### Bounding box generation and atlas selection for improving computational efficiency

Instead of applying the pattern recognition based label fuse to all voxels of the target image, we reduced the computational cost by following preprocessing steps. First, we linearly aligned all the images to the MNI152 template with voxel size of 1 × 1 × 1 *mm*^3^ and identified bounding boxes that were large enough for covering the hippocampal region in the MNI space (Hao et al., 2014). Second, we selected 20 most similar atlases for each target image by computing and ranking normalized mutual information between each candidate atlas image and the target image within the bounding box, and then registered the selected atlas images to the target image (Avants et al., 2009). Third, the majority voting label fusion method was used to obtain an initial segmentation result of the target image, and the RF-SSLP based label fusion was then applied to voxels without 100% votes for either the foreground or the background in the majority voting method (Hao et al., 2014).

### Parameter optimization based on the training data

Our method has tunable parameters for both the image feature extraction and the pattern recognition. For the image feature extraction, we followed the local label learning study to set the image patch size and the neighborhood size for training samples (Hao et al., 2014). Particularly, *r*_*s*_ = 3 for the image patches, and *r* = 1 for the neighborhood size. For the pattern recognition, our method has 6 parameters, including *k* (the number of nearest neighboring samples for training local random forests), *N*_*Tree*_ (the number of trees), *N*_*Split*_ (the number of predictors sampled for splitting at each node), *T* (the threshold for determining the reliable segmentation), σ (the image similarity parameter), and β (the trade-off parameter for updating the segmentation). Based on the training dataset, we adopted leave-one-out (LOO) cross-validation to optimize Dice index by grid-searching parameters from *k*∈{100, 200}, *N*_*Tree*_ ∈ {100, 200}, *N*_*Split*_ ∈ {10, 20, 30}, *T* ∈ {0.4, 0.5, 0.6}, σ ∈ {5, 10, 20}, and β ∈ {0.5, 0.6, 0.7}.

Particularly, the RF parameter optimization was implemented based on 12 settings of (*k* × *N*_*Tree*_ × *N*_*Split*_ = 2 × 2 × 3 = 12). For each setting of RF parameters, we optimized the SSLP parameter under 27 different settings of (*T* × σ × β = 3 × 3 × 3 = 27). The optimized parameters were finally determined by grid-searching all of the parameter combinations.

### Evaluation of segmentation accuracy

The evaluation of hippocampus segmentation was based on the training and testing data. Particularly, Dice, Jaccard, Precision, Recall, Mean Distance(MD), Harsdorff Distance(HD), Average Symmetric Surface Distance (ASSD) were computed to measure the differences between the manual segmentation label *A* and a result *B* obtained by an automatic image segmentation method, defined as (Hao et al., 2014)

$$\begin{array}{l} \text{Dice}=2\frac{\text{V}\left(\text{A}\cap \text{B}\right)}{\text{V}\left(\text{A}\right)+\text{V}\left(\text{B}\right)},\text{ Jaccard}=\frac{\text{V}\left(\text{A}\cap \text{B}\right)}{\text{V}\left(A\cup B\right)},\\ \text{Precision}=\frac{\text{V}\left(A\cap B\right)}{\text{V}\left(\text{B}\right)},\text{ Recall}=\frac{\text{V}\left(\text{A}\cap \text{B}\right)}{\text{V}\left(\text{A}\right)},\\ \text{MD}={\text{mean}}_{e\in \partial A}\left(\min_{f\in \partial B}d\left(e,f\right)\right),\\ \text{HD}=\max\left(\text{H}\left(A,\text{B}\right),H\left(\text{B},A\right)\right),\\ where\text{ H}\left(A,\text{B}\right)=\max_{e\in \partial A}\left(\min_{f\in \partial B}d\left(e,f\right)\right), \end{array}$$

HD95: similar to HD, except that 5% data points with the largest distance are removed before calculation,

$$\begin{array}{l} \text{ASSD}=({\text{mean}}_{e\in \partial A}\left(\min_{f\in \partial B}d\left(e,f\right)\right)\\ \;+{\text{mean}}_{e\in \partial B}\left(\min_{f\in \partial A}d\left(e,f\right)\right))/2,\\ \text{ RMSD}=\frac{\sqrt{D_{A}^{2}+D_{B}^{2}}}{card\left\{\partial A\right\}+card\left\{\partial B\right\}}, \end{array}$$

$$\text{RMSD}=\frac{\sqrt{D_{A}^{2}+D_{B}^{2}}}{card\left\{\partial A\right\}+card\left\{\partial B\right\}},$$

$$where\;D_{A}^{2}=\sum_{e\in \partial A}^{\;}\left(\underset{{\;}_{f\in \partial B}}{\min}d\left(e,f\right)\right).$$

### Comparison with alternative MAIS algorithms

We compared our method with alternative MAIS methods, including majority voting (MV) (Rohlfing et al., 2004), nonlocal patch (NLP) (Coupé et al., 2011), local label learning (LLL) (Hao et al., 2014), random local binary pattern (RLBP) (Zhu et al., 2015), and metric learning (ML) (Zhu et al., 2016, 2017). Parameters of these methods were set to optimal values suggested in their respective studies. For a fair comparison, we utilized the same parameters as recommended in Hao et al. (2014) with fixed parameters *r*_*s*_ = 3, *r* = 1 for patch size (2*r*_*s*_ + 1) × (2*r*_*s*_ + 1) × (2*r*_*s*_ + 1) and training sample neighborhood size (2*r* + 1) × (2*r* + 1) × (2*r* + 1) for all the image patch based label fusion methods.

Since the MV method could provide probabilistic segmentation results too, we integrated the same SSLP method with the MV method for the hippocampus segmentation, referred to as MV-SSLP. The same parameters used in our method were adopted in the MV-SSLP method.

For the NLP method (Coupé et al., 2011), the parameter σ in the Gauss similarity model was adaptively set to $\sigma =\underset{y\in N\left(x\right)}{\min}\left\{|P\left(x\right)-P\left(x_{s,j}\right){|}_{2}^{2}\;+\epsilon \right\}$ with ϵ = 1*e* − 20. For the LLL method (Hao et al., 2014), the number *k* of training samples was set to 400. For the RLBP method (Zhu et al., 2015), the dimension *L* of the generated RLBP feature was set to 1,000 and the balance parameter *C* was set to 4^−4^. For the ML method (Zhu et al., 2017), the *k* nearest training samples was set to 9.

## Experimental results

### Parameter optimization result based on the training data

Table 2 summarizes average Dice index measures of both the left and right hippocampal segmentation results of the training data obtained by the RF-based MAIS with 12 different settings of parameters from *k*∈{100, 200}, *N*_*Tree*_ ∈ {100, 200}, *N*_*Split*_ ∈ {10, 20, 30}, and followed by Dice index measures of segmentation results obtained by the SSLP method with optimized parameters by searching 27 different parameter settings of *T*∈{0.4, 0.5, 0.6}, σ ∈ {5, 10, 20}, and β ∈ {0.5, 0.6, 0.7}. These results demonstrated that the proposed RF-SSLP method obtained the best segmentation results (Dice index = 0.8904) based on the RF results obtained with *k* = 100, *N*_*Tree*_ = 200, and *N*_*Split*_ = 20.

**Table 2:** Average dice index of segmentation results obtained by RF based MAIS with *k* ∈ {100, 200}, *N*_*Tree*_ ∈ {100, 200}, and *N*_*Split*_ ∈ {10, 20, 30}, followed by optimized SSLP results with *T* ∈ {0.4, 0.5, 0.6}, σ ∈ {5, 10, 20}, and β ∈ {0.5, 0.6, 0.7}.

** 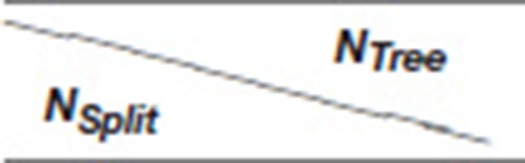 **		**100**	**200**
10	Left	0.8849/0.8901	0.8851/0.8896
	Right	0.8855/0.8903	0.8862/0.8905
20	Left	0.8844/0.8902	**0.8855/0.8902**
	Right	0.8853/0.8904	**0.8855/0.8907**
30	Left	0.8843/0.8901	0.8849/0.8902
	Right	0.8847/0.8903	0.8854/0.8906
*k* = 100			
10	left	0.8852/0.8893	0.8854/0.8894
	Right	0.8859/0.8898	0.8864/0.8899
20	Left	0.8847/0.8896	0.8858/0.8898
	Right	0.8858/0.8901	0.8861/0.8900
30	Left	0.8853/0.8895	0.8852/0.8897
	Right	0.8855/0.8898	0.8858/0.8902
*k* = 200			

*The bold values represent the best results*.

Table 3 summarizes average Dice index measures of both the left and right hippocampal segmentation results of the training data obtained by the SSLP by searching 27 different parameter settings based on the RF based segmentation results with *k* = 100, *N*_*Tree*_ = 200, and *N*_*Split*_ = 20. The results in Table 3 indicate that the SSLP method with *T* = 0.5, σ = 10, and β = 0.6 obtained the best segmentation performance. Therefore, the optimal parameters for our method were *k* = 100, *N*_*Tree*_ = 200, *N*_*Split*_ = 20, *T* = 0.5, σ = 10, and β = 0.6. These parameters were adopted in the following experiments.

**Table 3:** Average dice index of segmentation results obtained by the SSLP with *T* ∈ {0.4, 0.5, 0.6}, σ ∈ {5, 10, 20}, and β ∈ {0.5, 0.6, 0.7} based on the best segmentation performance selected by **Table 2**.

** 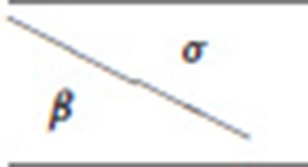 **				
		**5**	**10**	**20**
0.5	Left	0.8890	0.8897	0.8892
	Right	0.8894	0.8902	0.8901
0.6	Left	0.8889	0.8901	0.8894
	Right	0.8892	0.8906	0.8904
0.7	Left	0.8882	0.8898	0.8890
	Right	0.8877	0.8899	0.8899
*T* = 0.4				
0.5	Left	0.8890	0.8894	0.8888
	Right	0.8894	0.8901	0.8897
0.6	Left	0.8891	**0.8901**	0.8893
	Right	0.8894	**0.8907**	0.8905
0.7	Left	0.8884	0.8902	0.8897
	Right	0.8886	0.8905	0.8906
*T* = 0.5				
0.5	Left	0.8887	0.8891	0.8886
	Right	0.8891	0.8896	0.8893
0.6	Left	0.8888	0.8896	0.8890
	Right	0.8892	0.8903	0.8900
0.7	Left	0.8883	0.8899	0.8892
	Right	0.8887	0.8903	0.8901
*T* = 0.6				

*The bold values represent the best results*.

Table 4 summarizes the best segmentation performance of the MV, MV-SSLP, RF classification, and RF-SSLP on the training data, gaged by Dice index measures of both the left and right hippocampi estimated using a LOO cross-validation. These results demonstrated that the SSLP method could improve segmentation results of both the MV and the RF label fusion methods, and the integration of RF classification and SSLP obtained the best performance.

**Table 4:** Dice index values (mean ± std) for 35 training subjects.

	**Left hippocampus**	**Right hippocampus**
MV	0.8592 ± 0.03	0.8633 ± 0.02
MV-SSLP	0.8673 ± 0.03	0.8712 ± 0.02
RF	0.8850 ± 0.02	0.8857 ± 0.01
RF-SSLP	0.8898 ± 0.02	0.8908 ± 0.01

### Comparison with alternative MAIS methods based on the testing data

**Figure 2:**
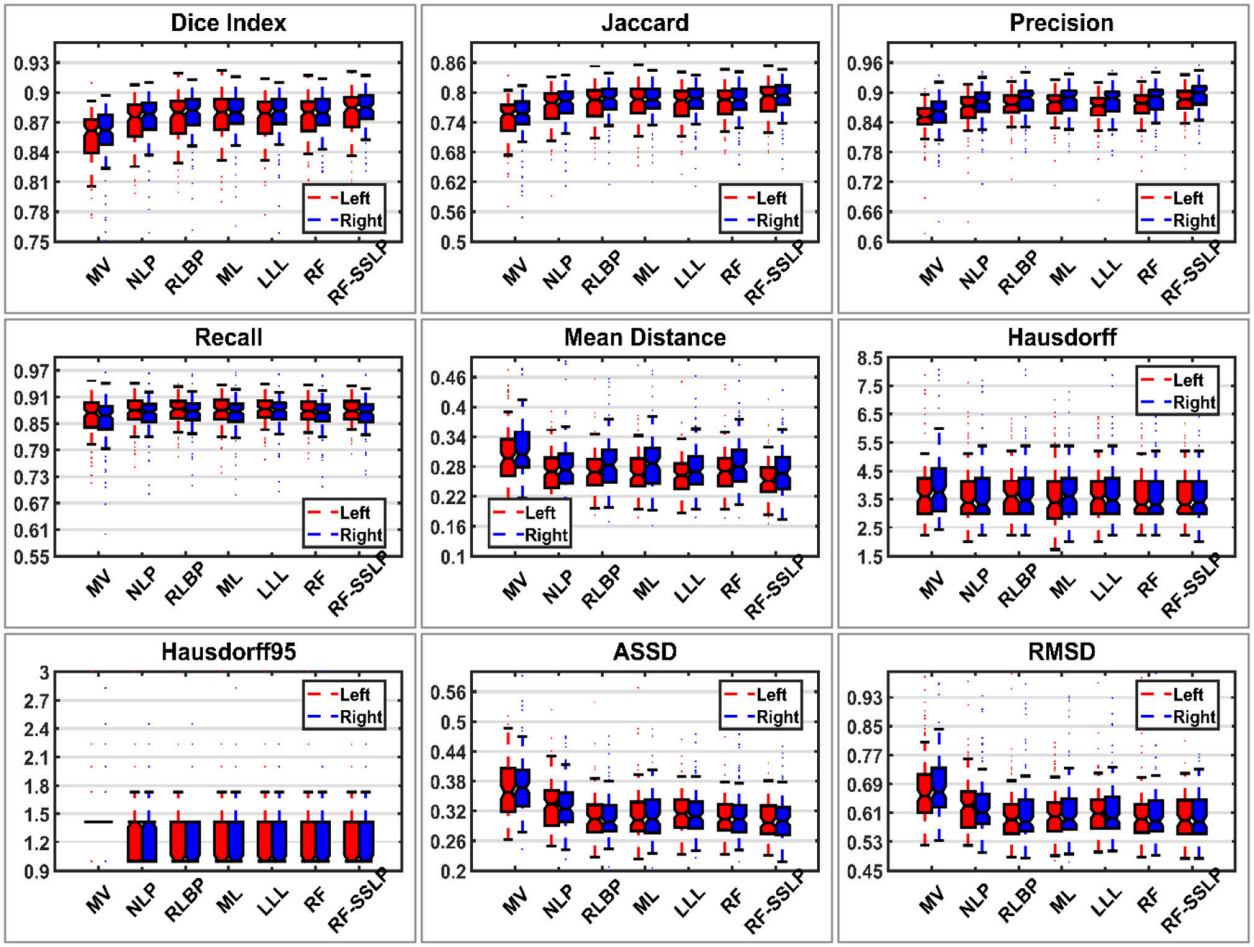
Box plots of segmentation performance of the methods under comparison based on testing data, evaluated based on nine different metrics. In each box, the central mark is the median and edges are the 25 and 75th percentiles.

Table 5 summarizes performance of the segmentation methods under comparison on the testing dataset. Wilcoxon signed rank tests were adopted to compare the methods under comparison in terms of 9 segmentation accuracy metrics. The statistical significance testing results indicated that our method performed better than the MV, NLP, RLBP, ML, LLL, RF classification methods with respect to most of the metrics (*p* < 0.05). Figure 2 shows box plots of segmentation performance of the methods under comparison based on the testing data, evaluated based on 9 different metrics.

**Table 5:** Nine index values (mean ± std) for hippocampus segmentation evaluation using different label fusion methods on testing data (^*^ indicates that our method achieved significantly superior results in the Wilcoxon signed rand tests with *p* < 0.05).

		**MV**	**NLP**	**RLBP**	**ML**	**LLL**	**RF**	**RF-SSLP**
Dice	Left	0.8552 ± 0.02^*^	0.8704 ± 0.02^*^	0.8757 ± 0.02^*^	0.8757 ± 0.02^*^	0.8744 ± 0.02^*^	0.8754 ± 0.02^*^	**0.8801 ± 0.01**
	Right	0.8555 ± 0.03^*^	0.8731 ± 0.02^*^	0.8768 ± 0.02^*^	0.8771 ± 0.02^*^	0.8769 ± 0.02^*^	0.8767 ± 0.02^*^	**0.8814 ± 0.01**
Jaccard	Left	0.7481 ± 0.04^*^	0.7714 ± 0.03^*^	0.7795 ± 0.03^*^	0.7797 ± 0.03^*^	0.7775 ± 0.03^*^	0.7790 ± 0.03^*^	**0.7866 ± 0.03**
	Right	0.7488 ± 0.04^*^	0.7757 ± 0.03^*^	0.7814 ± 0.03^*^	0.7819 ± 0.03^*^	0.7815 ± 0.03^*^	0.7813 ± 0.03^*^	**0.7886 ± 0.02**
Precision	Left	0.8457 ± 0.04^*^	0.8656 ± 0.03^*^	0.8716 ± 0.03^*^	0.8745 ± 0.03^*^	0.8685 ± 0.03^*^	0.8741 ± 0.03^*^	**0.8821 ± 0.03**
	Right	0.8559 ± 0.04^*^	0.8771 ± 0.03^*^	0.8824 ± 0.03^*^	0.8850 ± 0.03^*^	0.8811 ± 0.03^*^	0.8846 ± 0.03^*^	**0.8935 ± 0.03**
Recall	Left	0.8675 ± 0.04^*^	0.8770 ± 0.03	0.8809 ± 0.02^*^	0.8782 ± 0.03	0.8817 ± 0.03	0.8777 ± 0.02	**0.8793 ± 0.03**
	Right	0.8588 ± 0.05^*^	0.8712 ± 0.04	0.8730 ± 0.03^*^	0.8712 ± 0.04	0.8744 ± 0.03	0.8705 ± 0.03	**0.8711 ± 0.03**
MD	Left	0.3033 ± 0.05^*^	0.2699 ± 0.04^*^	0.2702 ± 0.04^*^	0.2711 ± 0.05^*^	0.2630 ± 0.03^*^	0.2704 ± 0.03^*^	**0.2540 ± 0.04**
	Right	0.3196 ± 0.07^*^	0.2780 ± 0.05^*^	0.2895 ± 0.06^*^	0.2871 ± 0.06^*^	0.2786 ± 0.05^*^	0.2885 ± 0.05^*^	**0.2702 ± 0.05**
HD	Left	3.8109 ± 1.13	3.6028 ± 1.04	3.6669 ± 0.99	3.6077 ± 1.08	3.6531 ± 1.02	3.6068 ± 1.01	**3.5605 ± 1.03**
	Right	4.0700 ± 1.39^*^	3.6949 ± 1.14	3.7108 ± 1.11	3.7232 ± 1.10	3.7116 ± 1.16	3.6845 ± 1.15	**3.6442 ± 0.61**
HD95	Left	1.4926 ± 0.49^*^	1.3584 ± 0.47^*^	1.1880 ± 0.35	1.2224 ± 0.44	1.2787 ± 0.39	**1.1813 ± 0.34**	1.2511 ± 0.26
	Right	1.5506 ± 0.64^*^	1.3501 ± 0.41^*^	**1.1912 ± 0.32**	1.2064 ± 0.37	1.2303 ± 0.33	1.2011 ± 0.33	1.2165 ± 0.20
ASSD	Left	0.3715 ± 0.07^*^	0.3347 ± 0.06^*^	0.3072 ± 0.04	0.3123 ± 0.05^*^	0.3189 ± 0.05^*^	0.3091 ± 0.04^*^	**0.3062 ± 0.04**
	Right	0.3799 ± 0.08^*^	0.3324 ± 0.05^*^	0.3092 ± 0.04	0.3144 ± 0.04^*^	0.3168 ± 0.04^*^	0.3116 ± 0.04^*^	**0.3065 ± 0.04**
RMSD	Left	0.6860 ± 0.12^*^	0.6406 ± 0.11^*^	**0.6067 ± 0.08**	0.6163 ± 0.10	0.6213 ± 0.08^*^	0.6074 ± 0.08	0.6070 ± 0.08
	Right	0.7055 ± 0.16^*^	0.6394 ± 0.10^*^	0.6133 ± 0.09	0.6198 ± 0.09^*^	0.6213 ± 0.08^*^	0.6143 ± 0.09	**0.6081 ± 0.05**

*The bold values represent the best results*.

**Figure 3:**
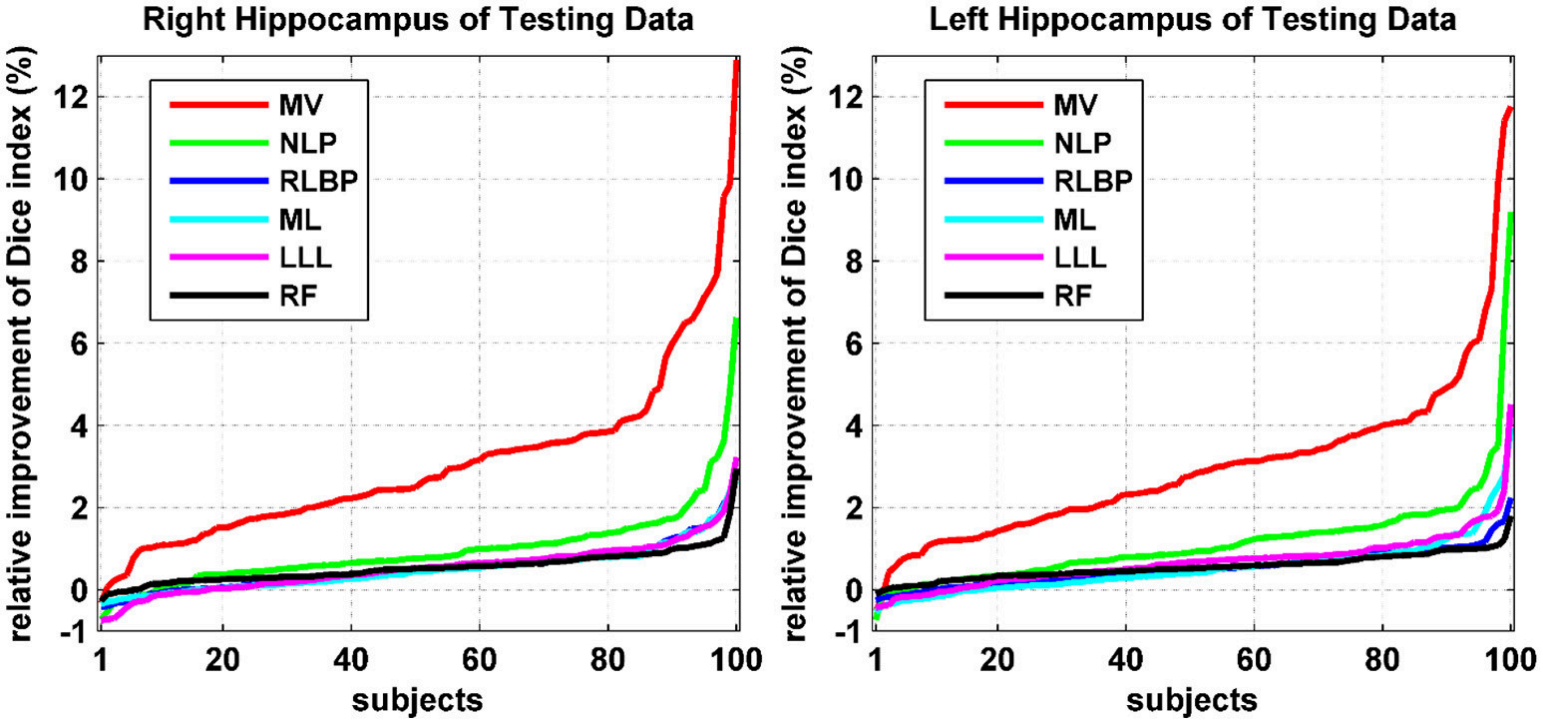
Relative improvement (%) achieved by our method compared with alternative state-of-the-art techniques in terms of Dice index values of individual testing images. The relative improvement rates of individual testing images were ranked separately for different methods.

Relative improvement achieved by our method was measured in terms of Dice index values of individual testing images. Particularly, the relative improvement rate of each individual image was calculated as the difference between Dice index values obtained by our method and an alternative method divided by the Dice index value obtained by the alternative method. As shown in Figure 3, our method consistently improved the segmentation accuracy in most cases by up to 13%. For some cases, our method had relatively worse segmentation performance than the alternative techniques under comparison. However, the degradation was <1% for all these cases. These results further demonstrated that our method could improve the overall hippocampus segmentation performance.

All the segmentation results indicated that the MV method obtained the overall worst segmentation performance and the RF-SSLP obtained the overall best segmentation performance. We inspected the worst segmentation results obtained by these two methods. For the left hippocampus, according to Dice index measures, the MV had the worst segmentation result for the subject “098-S-0172,” the RF-SSLP obtained the worst segmentation results for the subject “100-S-0995,” and the MV and the RF-SSLP obtained segmentation results with the largest difference for the subject “073-S-0089.” For the right hippocampus, according to Dice index measures, both the MV and the RF-SSLP obtained the worst segmentation results for the subject “016-S-0991,” and they obtained segmentation results with the largest difference for the subject “123-S-0091.” We also found that the RF-SSLP improved the RF with respect to Dice index for segmenting the left hippocampus of the subject “082-S-1079.” Table 6 summarizes segmentation performance obtained by the methods under comparison for these subjects.

**Table 6:** Dice index values of selected scans obtained by different methods under comparison.

**Subject ID**	**MV**	**NLP**	**RLBP**	**ML**	**LLL**	**RF**	**RF-SSLP**	**Description**
**LEFT HIPPOCAMPUS**								
098-S-0172	0.7266	0.7437	0.7992	0.7900	0.7770	0.8113	0.8121	Subject with the most inaccurate result by MV method.
100-S-0995	0.8054	0.8066	0.8085	0.8025	0.8100	0.8077	0.8088	Subject with the most inaccurate result by our method RF-SSLP.
073-S-0089	0.7772	0.8122	0.8471	0.8448	0.8455	0.8610	0.8660	Subject with the biggest difference between MV and our method RF-SSLP.
082-S-1079	0.7935	0.8258	0.8350	0.8098	0.8327	0.8269	0.8417	Subject with the biggest difference between RF and our method RF-SSLP.
**RIGHT HIPPOCAMPUS**								
016-S-0991	0.7086	0.7591	0.7616	0.7656	0.7587	0.7609	0.7852	Subject with the most inaccurate result by both MV and our method, and also with the biggest difference between RF and our method RF-SSLP.
123-S-0091	0.7442	0.7947	0.8320	0.8288	0.8316	0.8469	0.8526	Subject with the biggest difference between MV and our method RF-SSLP.

**Figure 4:**
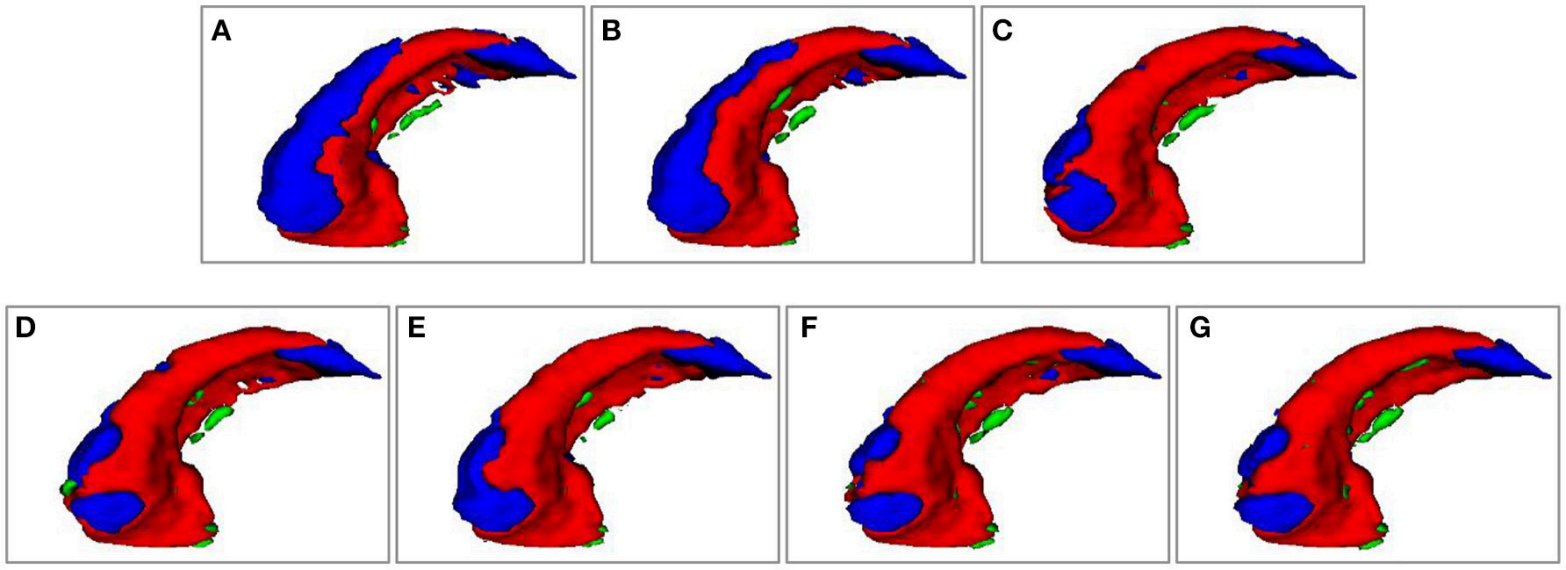
Visualization of segmenation resutls of the right hippocampus of subject “123-S-0091,” obtained by the segmenation methods under comparison. **(A)** MV; **(B)** NLP; **(C)** RLBP; **(D)** ML; **(E)** LLL; **(F)** RF; **(G)** RF-SSLP (Red: overlap between manual and segmentation results. Blue: manual results. Green: segmentation results).

**Figure 5:**
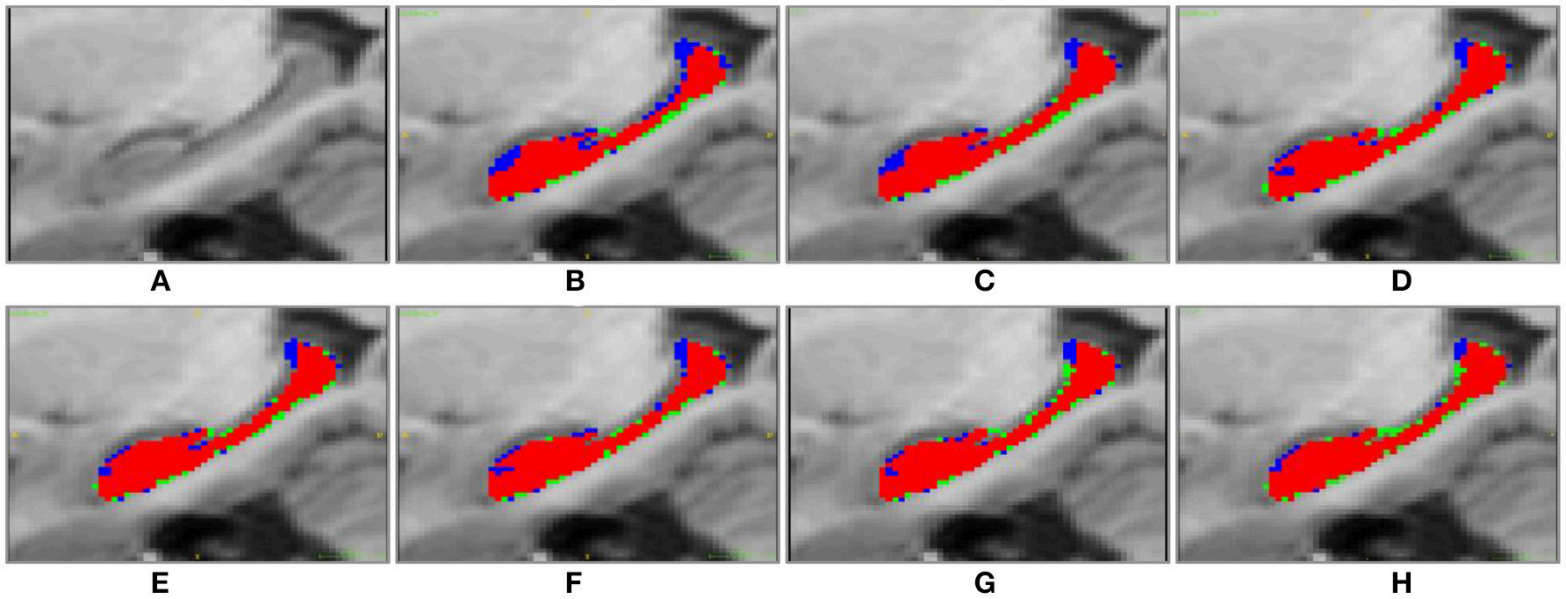
Sagittal visulization of segmenation resutls of the right hippocampus of subject “123-S-0091,” obtained by the segmenation methods under comparison. **(A)** original image; **(B)** MV; **(C)** NLP; **(D)** RLBP; **(E)** ML; **(F)** LLL; **(G)** RF; **(H)** RF-SSLP (Red: overlap between manual and segmentation results. Blue: manual results. Green: segmentation results).

**Figure 6:**
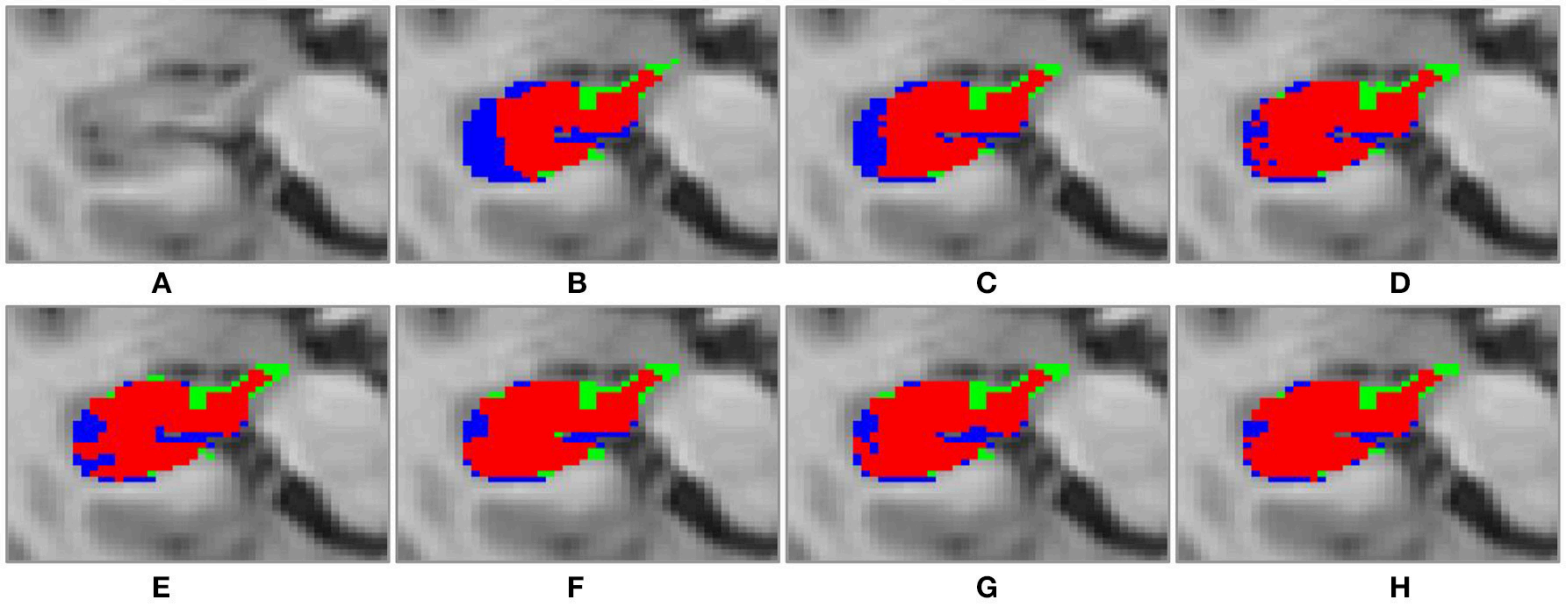
Transverse visulization of segmenation resutls of the right hippocampus of subject “123-S-0091,” obtained by the segmenation methods under comparison. **(A)** original image; **(B)** MV; **(C)** NLP; **(D)** RLBP; **(E)** ML; **(F)** LLL; **(G)** RF; **(H)** RF-SSLP (Red: overlap between manual and segmentation results. Blue: manual results. Green: segmentation results).

Figure 4 shows 3D visualization results of the right hippocampus of the subject “123-S-0091” segmented by the segmentation methods under comparison, and Figures 5, 6 show their 2D visualization results. These results demonstrated that more sophisticated label fusion methods could improve the segmentation performance over the simple MV method, and the RF-SSLP method could improve the label fusion by exploiting the voxel correlation information in the target image.

## Discussion and conclusions

We proposed a MAIS method by integrating the random forests based multi-atlas segmentation and the semi-supervised label propagation under the multi-atlas based image segmentation framework. Experiment results for segmenting the hippocampus from MRI scans based on the EADC-ADNI dataset demonstrated that our method achieved competitive hippocampus segmentation performance compared with alternative methods under comparison. We have made source codes of the methods under comparison publicly available. These source codes can not only be used as benchmark methods for comparing MAIS methods, but also be useful for segmenting and quantifying hippocampus in imaging studies.

The MAIS methods have achieved promising performance in a variety of image segmentation studies partially due to their capability to incorporate shape information of structures to be segmented through registering multiple atlases to images to be segmented. However, most of the existing MAIS methods typically fuse the registered label information of different voxels, ignoring potential correlations among voxels in the target image. The proposed RF-SSLP method integrates a semi-supervised label propagation method and a supervised random forests method in the MAIS framework to segment the target image by propagating reliable segmentation information within the target image regularized by local and global image consistency. The experimental results have demonstrated that the RF-SSLP could improve the segmentation, indicating that the voxel correlation information in the target image could help improve the image segmentation performance.

The results summarized in Table 6 also indicated that for some images, such as 016-S-0991, all the segmentation methods under comparison had limited performance, although the proposed RF-SSLP method still had the best performance evaluated based on Dice index measures. The relatively poor segmentation performance of MAIS methods might be caused by large anatomical difference between images to be segmented and the atlas images. To solve this problem, besides improving the image registration (Li and Fan, 2017, 2018), we could obtain a large number of atlas images so that the images to be segmented could have a larger chance of being well-aligned with the atlas images. In the present study, a fixed number of atlas images were selected to obtain the image segmentation results. The image segmentation performance might be improved if the atlas images most similar to images to be segmented are adaptively selected, for instance selecting atlas images according to an image similarity threshold. Furthermore, deep learning techniques could also be adopted to improve the image segmentation if a large number of training data are available (Zhao et al., 2018).

In conclusion, the RF-SSLP method could obtain competitive image segmentation performance compared with alternative MAIS methods under comparison. The improved performance obtained by the RF-SSLP method can be attributed to taking into consideration correlation among voxels of images to be segmented in the label fusion. A better atlas selection method capable of adaptively selected atlases might be needed to further improve the segmentation performance of MAIS methods in addition to improve the image registration performance.

## Author contributions

All the authors contributed to the method development. QZ undertook the statistical analysis. All authors contributed to the manuscript preparation.

### Conflict of interest statement

The authors declare that the research was conducted in the absence of any commercial or financial relationships that could be construed as a potential conflict of interest.

## References

[B1] Alchatzidis S. Sotiras A. Zacharaki E. I. Paragios N. (2017). A discrete MRF framework for integrated multi-atlas registration and segmentation. Int. J. Comput. Vis. 121, 169–181. 10.1007/s11263-016-0925-2

[B2] Alven J. Norlen A. Enqvist O. Kahl F. (2016). Uberatlas: fast and robust registration for multi-atlas segmentation. Pattern Recognit. Lett. 80, 249–255. 10.1016/j.patrec.2016.05.001

[B3] Amoroso N. Errico R. Bruno S. Chincarini A. Garuccio E. Sensi F.. (2015). Hippocampal unified multi-atlas network (HUMAN): protocol and scale validation of novel segmentation tool. Phys. Med. Biol. 60, 8851–8867. 10.1088/0031-9155/60/22/8851 26531765

[B4] Avants B. B. Tustison N. J. Song G. (2009). Advanced normalization tools (ANTS). Insight J. 2, 1–35.

[B5] Boccardi M. Bocchetta M. Morency F. C. Collins D. L. Nishikawa M. Ganzola R.. (2015). Training labels for hippocampal segmentation based on the EADC-ADNI harmonized hippocampal protocol. Alzheimers Dementia 11, 175–183. 10.1016/j.jalz.2014.12.002 25616957

[B6] Breiman L. (2001). Random forests. Mach. Learn. 45, 5–32. 10.1023/A:1010933404324

[B7] Coupé P. Manjón J. V. Fonov V. Pruessner J. Robles M. Collins D. L. (2011). Patch-based segmentation using expert priors: application to hippocampus and ventricle segmentation. Neuroimage 54, 940–954. 10.1016/j.neuroimage.2010.09.018 20851199

[B8] Doshi J. Erus G. Ou Y. M. Resnick S. M. Gur R. C. Gur R. E.. (2016). MUSE: multi-atlas region segmentation utilizing ensembles of registration algorithms and parameters, and locally optimal atlas selection. Neuroimage 127, 186–195. 10.1016/j.neuroimage.2015.11.073 26679328 PMC4806537

[B9] Giraud R. Ta V. T. Papadakis N. Manjon J. V. Collins D. L. Coupe P. (2016). An optimized patch match for multi-scale and multi-feature label fusion. Neuroimage 124, 770–782. 10.1016/j.neuroimage.2015.07.076 26244277

[B10] Han X. (2013). Learning-boosted label fusion for multi-atlas auto-segmentation. Machine Learn. Med. Image 8184, 17–24. 10.1007/978-3-319-02267-3_3

[B11] Hao Y. Jiang T. Fan Y. (2012a). “Iterative multi-atlas based segmentation with multi-channel image registration and Jackknife Context Model,” in 2012 9th IEEE International Symposium on Biomedical Imaging (ISBI) (Barcelona), 900–903.

[B12] Hao Y. Jiang T. Fan Y. (2012b). “Shape-constrained multi-atlas based segmentation with multichannel registration,” in Proceeding of SPIE Medical Imaging: Image Processing 8314, 83143N (San Diego, CA).

[B13] Hao Y. Liu J. Duan Y. Zhang X. Yu C. Jiang T. (2012c). “Local label learning (L3) for multi-atlas based segmentation,” in SPIE Medical Imaging: SPIE, 83142E (San Diego, CA).

[B14] Hao Y. Wang T. Zhang X. Duan Y. Yu C. Jiang T.. (2014). Local label learning (LLL) for subcortical structure segmentation: application to hippocampus segmentation. Hum. Brain Mapp. 35, 2674–2697. 10.1002/hbm.22359 24151008 PMC6869539

[B15] Jack C. R. Bernstein M. A. Fox N. C. Thompson P. Alexander G. Harvey D.. (2008). The Alzheimer's disease neuroimaging initiative (ADNI): MRI methods. J. Magn. Reson. Imaging 27, 685–691. 10.1002/jmri.21049 18302232 PMC2544629

[B16] Koch L. M. Wright R. Vatansever D. Kyriakopoulou V. Malamateniou C. Patkee P. A. (2014). Graph-based label propagation in fetal brain MR images. Int. Workshop Machine Learn. Med. Imaging (MLMI) 8679, 9–16. 10.1007/978-3-319-10581-9_2

[B17] Li H. Fan Y. (2012). “Label propagation with robust initialization for brain tumor segmentation,” in IEEE International Symposium on Biomedical Imaging (ISBI) (Barcelona), 1715–1718.

[B18] Li H. Fan Y. (2017). Non-Rigid Image Registration Using Fully Convolutional Networks with Deep Self-Supervision arXiv: 1709.00799v1.

[B19] Li H. Fan Y. (2018). “Non-Rigid Image Registration Using Self-Supervised Fully Convolutional Networks without Training Data,” in IEEE International Symposium on Biomedical Imaging (ISBI) (Washington, DC), 1–4. 10.1109/ISBI.2018.8363757 PMC6070305 30079127

[B20] Li H. Song M. Fan Y. (2010). “Segmentation of brain tumors in multi-parametric MR images via robust statistic information propagation,” in Asian Conference on Computer Vision (ACCV), (Queenstown) 606–61.

[B21] Rohlfing T. Brandt R. Menzel R. Maurer C. R. Jr (2004). Evaluation of atlas selection strategies for atlas-based image segmentation with application to confocal microscopy images of bee brains. Neuroimage 21, 1428–1442. 10.1016/j.neuroimage.2003.11.010 15050568

[B22] Roy S. He Q. Sweeney E. Carass A. Reich D. S. Prince J. L.. (2015). Subject-specific sparse dictionary learning for atlas-based brain MRI segmentation. IEEE J. Biomed. Health Inform. 19, 1598–1609. 10.1109/JBHI.2015.2439242 26340685 PMC4562064

[B23] Wang H. Suh J. W. Das S. R. Pluta J. B. Craige C. Yushkevich P. A. (2013). Multi-atlas segmentation with joint label fusion. IEEE Trans. Pattern Anal. Mach. Intell. 35, 611–623. 10.1109/TPAMI.2012.143 22732662 PMC3864549

[B24] Wu G. Kim M. Sanroma G. Wang Q. Munsell B. C. Shen D. G. (2015). Hierarchical multi-atlas label fusion with multi-scale feature representation and label-specific patch partition. Neuroimage 106, 34–46. 10.1016/j.neuroimage.2014.11.025 25463474 PMC4285661

[B25] Yang X. Fan Y. (2018a). “Coupled dictionary learning for joint MR image restoration and segmentation,” in SPIE Medical Imaging: SPIE (Houston, TX), 8.

[B26] Yang X. Fan Y. (2018b). “Feature extraction using convolutional neural networks for multi-atlas based image segmentation,” in SPIE Medical Imaging: SPIE (Houston, TX), 8.

[B27] Zhang L. Wang Q. Gao Y. Z. Li H. X. Wu G. R. Shen D. G. (2017). Concatenated spatially-localized random forests for hippocampus labeling in adult and infant MR brain images. Neurocomputing 229, 3–12. 10.1016/j.neucom.2016.05.082 28133417 PMC5268165

[B28] Zhao X. M. Wu Y. H. Song G. D. Li Z. Y. Zhang Y. Z. Fan Y. (2018). A deep learning model integrating FCNNs and CRFs for brain tumor segmentation. Med. Image Anal. 43, 98–111. 10.1016/j.media.2017.10.002 29040911 PMC6029627

[B29] Zheng Q. Fan Y. (2018). “Integrating semi-supervised label propagation and random forests for multi-atlas based hippocampus segmentation,” in IEEE International Symposium on Biomedical Imaging (ISBI) (Washington, DC). 10.1109/ISBI.2018.8363544 PMC6070300 30079126

[B30] Zhou D. Y. Bousquet O. Lal T. N. Weston J. Scholkopf B. (2004). Learning with local and global consistency,” in 16th International Conference on Neural Information Processing Systems (NIPS) (Whistler, BC), 321–328.

[B31] Zhu H. Cheng H. Yang X. Fan Y. (2016). “Metric learning for label fusion in multi-atlas based image segmentation,” in 2016 IEEE 13th International Symposium on Biomedical Imaging (ISBI) (Prague), 1338–1341.

[B32] Zhu H. Cheng H. W. Yang X. S. Fan Y. (2017). Metric learning for multi-atlas based segmentation of hippocampus. Neuroinformatics 15, 41–50. 10.1007/s12021-016-9312-y 27638650 PMC5438876

[B33] Zhu H. C. Cheng H. W. Fan Y. (2015). “Random local binary pattern based label learning for multi-atlas segmentation,” in Processing of SPIE Medical Imaging: Image Processing 9413, 94131B (Orlando, FL).

[B34] Zu C. Wang Z. X. Zhang D. Q. Liang P. P. Shi Y. H. Shen D. G.. (2017). Robust multi-atlas label propagation by deep sparse representation. Pattern Recognit. 63, 511–517. 10.1016/j.patcog.2016.09.028 27942077 PMC5144541

